# Seven to Eight Hours of Sleep a Night Is Associated with a Lower Prevalence of the Metabolic Syndrome and Reduced Overall Cardiometabolic Risk in Adults

**DOI:** 10.1371/journal.pone.0072832

**Published:** 2013-09-05

**Authors:** Jean-Philippe Chaput, Jessica McNeil, Jean-Pierre Després, Claude Bouchard, Angelo Tremblay

**Affiliations:** 1 Healthy Active Living and Obesity Research Group, Children’s Hospital of Eastern Ontario Research Institute, Ottawa, Ontario, Canada; 2 Behavioural and Metabolic Research Unit, School of Human Kinetics, University of Ottawa, Ottawa, Ontario, Canada; 3 Department of Cardiology, Centre de recherche de l’Institut universitaire de cardiologie et de pneumologie de Québec, Quebec City, Quebec, Canada; 4 Department of Kinesiology, Faculty of Medicine, Laval University, Quebec City, Quebec, Canada; 5 Human Genomics Laboratory, Pennington Biomedical Research Center, Baton Rouge, Louisiana, United States of America; Simon Fraser University, Canada

## Abstract

**Background:**

Previous studies looking at the relationship between sleep duration and the metabolic syndrome have only used a dichotomous approach (presence/absence) and failed to adjust for important confounding factors. The objective of the present study was to examine the association between self-reported sleep duration and features of the metabolic syndrome in adults.

**Methods:**

A cross-sectional analysis from the Quebec Family Study (Canada) was conducted on 810 participants aged 18 to 65 years. Participants were categorized as short (≤6 h), adequate (7–8 h) or long (≥9 h) sleepers. The metabolic syndrome was defined according to the American Heart Association/National Heart, Lung, and Blood Institute’s criteria.

**Results:**

Overall, 24.6% of the sample had the metabolic syndrome. A U-shaped relationship between sleep duration and the prevalence of metabolic syndrome (33.3%, 22.0% and 28.8% in short, adequate and long sleepers, respectively) was observed (*P*<0.01). Only short sleepers had a significant increase in the odds of having the metabolic syndrome (OR = 1.76, 95% CI = 1.08–2.84) compared to adequate sleepers after adjustment for age, sex, smoking habits, highest education level, total annual family income, alcohol consumption, coffee intake, menopausal status, daily caloric intake, and moderate-to-vigorous physical activity. Likewise, the clustered cardiometabolic risk score (i.e. continuous risk score based on the metabolic syndrome components) was significantly higher in short sleepers compared to adequate sleepers after adjustment for covariates (*P*<0.05).

**Conclusion:**

Sleeping ≤6 h per night is associated with an elevated cardiometabolic risk score and an increase in the odds of having the metabolic syndrome after adjusting for possible confounders. These results strongly suggest that short sleep duration is a risk factor for the metabolic syndrome.

## Introduction

Insufficient sleep has become pervasive in modern societies, especially in children and adolescents, with 24/7 availability of commodities [1], [2]. Lack of sufficient sleep has been shown to exert deleterious effects on a variety of systems including detectable hormonal perturbations that may adversely impact health [3], [4]. Too little or too much sleep have been associated with obesity [5], [6], type 2 diabetes [7], [8], coronary heart disease [9], [10], hypertension [11], [12], and premature death [13], [14]. The association between sleep duration and many health outcomes appears to be U-shaped, suggesting that different mechanisms may operate at either end of the sleep duration distribution [15].

The metabolic syndrome, a cluster of cardiometabolic risk factors that predispose individuals to cardiovascular disease and type 2 diabetes, is a critical public health problem nowadays [16]. Sleep is intricately linked to various hormonal and metabolic systems and is likely to influence the development of the metabolic syndrome [17]. A small number of studies have recently shown that sleep duration is associated with the metabolic syndrome [18]–[22]. However, previous studies only looked at the association between sleep duration and the metabolic syndrome as a dichotomous diagnosis (presence/absence), and none examined the relationship with a clustered cardiometabolic risk score. The clustered risk score approach is used as a means of estimating an individual’s global cardiometabolic risk and results in a continuous risk score that increases statistical power [23]–[25].

The main objective of this observational, cross-sectional study was to investigate the relationship between sleep duration and features of the metabolic syndrome in adults from the greater Quebec City area (Canada). It was hypothesized that both short sleepers (≤6 h per night) and long sleepers (≥9 h per night) would be more likely to have features of the metabolic syndrome than those sleeping 7 to 8 hours a day, independent of relevant covariates.

## Methods

### Ethics Statement

All participants provided written informed consent to participate in the study. The project followed guidelines of the Medical Research Council of Canada, and was approved by the Medical Ethics Committee of Laval University (Quebec City, Canada).

### Participants

The Quebec Family Study was initiated at Laval University in 1978. The primary objective of this study was to investigate the role of genetics in the etiology of obesity and related cardiovascular risk factors. In phase 1 of the study (1978 to 1981), a total of 1650 individuals from 375 families were recruited and assessed. Recruitment was conducted irrespective of body weight during phase 1, resulting in a cohort with a wide range of body mass index, ranging from 13.8 to 64.9 kg/m^2^. In phase 2 (1989–1994) and 3 (1995–2001), 100 families from phase 1 were retested, and an additional 123 families with at least 1 parent and 1 offspring with a body mass index of 32 kg/m^2^ or higher were added to the cohort. Families were recruited through the media and were all French Canadians from the greater Quebec City area. Details of recruitment procedures have been published elsewhere [26]. This cohort thus represents a mixture of random sampling and ascertainment through obese individuals. The present analyses are based on participants tested in phases 2 and 3 because some measurements were not available in phase 1. Adults between 18 and 65 years of age were selected for cross-sectional analyses (*n* = 810).

The Quebec Family Study database is not publicly available. However, it has been used repeatedly over the last 30 years in a large number of collaborative studies. Investigators who are interested in using some of the data need to contact one of the senior investigators (Claude Bouchard, Angelo Tremblay or Jean-Pierre Després).

### Sleep Duration Assessment

The number of hours of sleep was assessed with a question that was inserted into a self-administered questionnaire on physical activity participation. The question was formulated as follows: “On average, how many hours do you sleep a day?” The participants were classified into 3 sleep duration groups: short sleepers (≤6 h of sleep), adequate sleepers (7–8 h of sleep) and long sleepers (≥9 h of sleep), in agreement with recent papers [27], [28]. The participants were classified into 3 sleep duration groups because of the U-shaped relationship that has been previously noted between sleep duration and most health outcomes. Epidemiological evidence indicates that a daily sleep duration of 7–8 h is optimal, and is associated with an overall good health status in adults [29].

### Features of the Metabolic Syndrome and Cardiometabolic Risk Score

Components of the metabolic syndrome were assessed in the morning following a 12-h overnight fast. Systolic (SBP) and diastolic (DBP) blood pressure measurements were obtained by trained staff, using a mercury sphygmomanometer and cuff size appropriate to the participant’s arm circumference. Blood pressure was calculated as the mean of two consecutive readings obtained on the right arm, in a seated position, and following 10 minutes of rest. Body weight, height and waist circumference (WC) (midpoint of the lowest rib and iliac crest) were measured according to standardized procedures [30], and body mass index (BMI) was calculated as body weight divided by height squared (kg/m^2^). Study staff also collected a venous blood sample to measure fasting serum lipids, glucose, and insulin concentrations, as previously described [27]. The metabolic syndrome was defined according to the American Heart Association/National Heart, Lung, and Blood Institute’s criteria [31] as the presence of three or more of the following risk factors: (i) waist circumference greater than 102 cm in men or greater than 88 cm in women; (ii) fasting serum glucose of 5.6 mmol/L or greater, or the use of oral hypoglycemic medication; (iii) systolic and diastolic blood pressures of at least 130 mm Hg and 85 mm Hg, respectively, or the use of antihypertensive medication; (iv) serum triglyceride levels of 1.7 mmol/L or higher, or the use of medication for hypertriglyceridemia; and (v) high-density lipoprotein (HDL) cholesterol levels of less than 1.03 mmol/L in men or 1.29 mmol/L in women, or the use of medication for low HDL cholesterol. Furthermore, a clustered cardiometabolic risk score was calculated for each participant by adding up Z scores for each variable as follows:

Clustered Cardiometabolic Risk Score = −zHDL+zInsulin+zGlucose+zTriglycerides+(zBMI+zWC)/2+ (zSBP+zDBP)/2.

This clustered risk score was used as a means of estimating an individual’s global cardiometabolic risk. In contrast to a dichotomous metabolic syndrome diagnosis, this approach provides a continuous risk score that increases statistical power, and has been used successfully in several recent investigations [23]–[25].

### Covariates

Numerous covariates were measured via self-reported questionnaires and reviewed during an interview with the research staff. These questionnaires (including sleep) were filled out the same day as the metabolic syndrome measures were obtained. Covariates included age, sex, smoking habits (smoker or nonsmoker), highest education level (high school, pre-university level [*CEGEP* for Quebec], university), total annual family income (Canadian dollars per year), coffee intake (number of cups per day), and menopausal status (in menopause or not in menopause). Additionally, daily energy intake (kcal/day) and alcohol consumption (g/day) were assessed with a 3-day food record [32]. This method of dietary assessment has been shown to provide a relatively reliable measurement of food intake in this population [32]. Finally, daily physical activity level was evaluated with a 3-day physical activity diary, as previously described [33]. Moderate-to-vigorous physical activity participation over 3 days was used for statistical analyses (min/day). These covariates were chosen because of their association with sleep duration and/or the metabolic syndrome.

### Statistical Analysis

Since there was no statistically significant gender interaction between sleep duration and the outcome variable (i.e. metabolic syndrome), data for both sexes were combined to improve clarity and maximize power. Descriptive characteristics of participants by sleep duration group were compared by analysis of variance (continuous variables) and chi-squared test (categorical variables). A Tukey post-hoc test was used to contrast mean differences. Logistic regression analysis was used to predict the odds of having the metabolic syndrome in short and long sleepers. The 7–8 h category was used as the reference group. The model was adjusted for age, sex, smoking habits, highest education level, total annual family income, alcohol consumption, coffee intake, menopausal status, daily caloric intake, and moderate-to-vigorous physical activity participation. Odds ratios (OR) and 95% confidence intervals (CI) were reported. Finally, clustered cardiometabolic risk scores were compared between sleep duration groups using an analysis of covariance, followed by a Tukey post-hoc test to contrast mean differences. The model was adjusted for the above-mentioned covariates. A 2-tailed *P* value of less than 0.05 was the threshold to indicate statistical significance. All statistical analyses were performed using JMP version 10 (SAS Institute, Cary, NC).

## Results

Baseline characteristics of participants within each sleep duration group are shown in Table 1. Among the 810 participants of this study, all of them were Caucasians, 38 (4.7%) had diagnosed type 2 diabetes, and 95 (11.7%) had diagnosed hypertension. Short sleepers had a significantly higher BMI and waist circumference compared to those sleeping 7 to 8 h per night. Fasting insulin concentrations were significantly different across sleep duration groups in a U-shaped curve. A significantly higher proportion (32%) of short sleepers were smokers, compared to adequate (22%) and long (16%) sleepers. Individuals in the 7–8 h sleep category had a significantly higher total annual income and short sleepers reported drinking more coffee than the long sleepers. Finally, short sleepers engaged in more moderate-to-vigorous physical activity compared to the two other sleep categories.

**Table 1:** Baseline characteristics of participants according to sleep duration group.

	≤6 h pernight (*n* = 90)	7–8 h per night(*n* = 571)	≥9 h per night (*n* = 149)	*P*
Age (years)	40.8±13.3	40.0±14.0	39.8±15.2	0.86
Sex				
Men (%)	48	44	36	
Women (%)	52	56	64	0.26
Body mass index (kg/m^2^)	29.5±7.3*	26.8±7.0	28.6±9.3	<0.01
Waist circumference (cm)	93.1±18.6*	87.1±17.5	90.3±21.1	<0.01
Fasting glucose (mmol/L)	5.44±0.88	5.31±1.04	5.60±1.96	0.14
Fasting insulin (pmol/L)	86.1±60.5*	71.6±59.4	87.1±69.0*	0.01
Systolic blood pressure (mm Hg)	116±15	117±16	120±17	0.20
Diastolic blood pressure (mm Hg)	74±13	72±10	74±10	0.16
Fasting triglycerides (mmol/L)	1.61±0.92	1.52±1.55	1.53±0.85	0.95
HDL cholesterol (mmol/L)	1.18±0.34	1.23±0.32	1.25±0.33	0.48
Smoking habits				
Nonsmoker (%)	68	78	84	
Smoker (%)	32*†	22	16	0.03
Highest education level				
High school (%)	55	41	47	
Pre-university level (%)	29	35	33	
University (%)	16	24	20	0.06
Total annual family income ($C)	49,937±33,373*	56,284±28,806	47,738±26,447*	0.01
Alcohol consumption (g/day)	12.8±14.2	8.1±16.1	7.3±17.3	0.15
Coffee intake (cups/day)	3.00±2.28†	2.84±2.53	2.04±1.68	<0.01
Menopausal status				
In menopause (%)	15	20	24	
Not in menopause (%)	85	80	76	0.28
Energy intake (kcal/day)	2,350±692	2,362±716	2,291±691	0.76
MVPA (min/day)	39±66*†	24±37	19±31	<0.01

Abbreviations: HDL, high-density lipoprotein; MVPA, moderate-to-vigorous physical activity. Values are mean ± SD or *n* (%).

Statistical significance was assessed by analysis of variance with continuous variables and by a chi-squared test with categorical variables. A Tukey post-hoc test was used to contrast mean differences. *Significantly different from the 7–8 h sleep group. †Significantly different from the ≥9 h sleep group.

A comparison of the prevalence of the components and the number of metabolic abnormalities among participants with different sleep durations is shown in Table 2. Individuals sleeping 7 to 8 h per night had the lowest prevalence of abdominal adiposity and those sleeping less than 6 h per night the greatest prevalence of low HDL cholesterol concentrations. Overall, 199 participants (24.6% of the sample) had the metabolic syndrome. A U-shaped relationship between sleep duration and the prevalence of metabolic syndrome (33.3%, 22.0% and 28.8% in short, adequate and long sleepers, respectively) was observed (*P*<0.01).

**Table 2:** Proportion of participants in each sleep duration category who meet criteria for the metabolic syndrome.^1^

	≤6 h per night (*n* = 90)	7–8 h per night (*n* = 571)	≥9 h per night (*n* = 149)	*P*
Criteria	38.9*	27.3	34.9	0.03
Abdominal adiposity	29.2	23.4	24.8	0.54
Glucose	22.2	20.8	28.8	0.12
Blood pressure	34.4	29.3	31.1	0.61
Triglycerides	58.8*†	44.7	43.9	<0.01
HDL cholesterol				
Number of metabolic abnormalities				
None	23.3	33.2	29.5	0.13
One component	25.5	26.6	27.5	0.94
Two components	17.8	18.0	14.1	0.51
Three or more (metabolic syndrome)	33.3*	22.0	28.8	<0.01

1 Metabolic syndrome defined according to the American Heart Association/National Heart, Lung, and Blood Institute’s criteria (AHA/NHLBI) as the presence of three or more of the following: (1) waist circumference greater than 102 cm in men or greater than 88 cm in women; (2) fasting serum glucose of 5.6 mmol/L or greater, or use of oral hypoglycemic medication; (3) blood pressure of 130 mm Hg systolic, 85 mm Hg diastolic or higher, or use of antihypertensive medication; (4) serum triglycerides of 1.7 mmol/L or higher, or medication for hypertriglyceridemia; and (5) high-density lipoprotein (HDL) cholesterol of less than 1.03 mmol/L in men or 1.29 mmol/L in women, or use of medication for low HDL cholesterol.

Abbreviations: HDL, high-density lipoprotein. Data are presented as percentage. Statistical significance was assessed by a chi-squared test.

A post-hoc test was used to contrast differences between sleep duration groups. *Significantly different from the 7–8 h sleep group.

† Significantly different from the ≥9 h sleep group.

The results of the logistic regression analysis assessing the relationship between sleep duration and the metabolic syndrome are summarized in Table 3. Short sleepers had significantly greater odds of having the metabolic syndrome (OR = 1.76, 95% CI = 1.08–2.84) compared to those sleeping 7 to 8 h per night, after adjusting for covariates. However, long sleep duration was not associated with an increase in the odds of having the metabolic syndrome (either unadjusted or adjusted models).

**Table 3:** Multivariate associations between sleep duration and the metabolic syndrome.^1^

	≤6 h per night (*n* = 90)		7–8 h per night (*n* = 571)	≥9 hours per night (n = 149)	
	OR	95% CI	Reference	OR	95% CI
Model 1	2.02	1.32–3.23	1.00	1.46	0.95–2.14
Model 2	1.80	1.15–2.98	1.00	1.43	0.91–2.09
Model 3	1.76	1.08–2.84	1.00	1.35	0.83–1.99

1 Metabolic syndrome defined according to the American Heart Association/National Heart, Lung, and Blood Institute’s criteria [31].

Model 1: unadjusted odds ratio.

Model 2: adjusted for age, sex, smoking habits, highest education level, total annual family income, alcohol consumption, coffee intake, and menopausal status.

Model 3: adjusted for daily caloric intake and moderate-to-vigorous physical activity in addition to age, sex, smoking habits, highest education level, total annual family income, alcohol consumption, coffee intake, and menopausal status.

Abbreviations: OR, odds ratio; CI, confidence interval.

**Figure 1:**
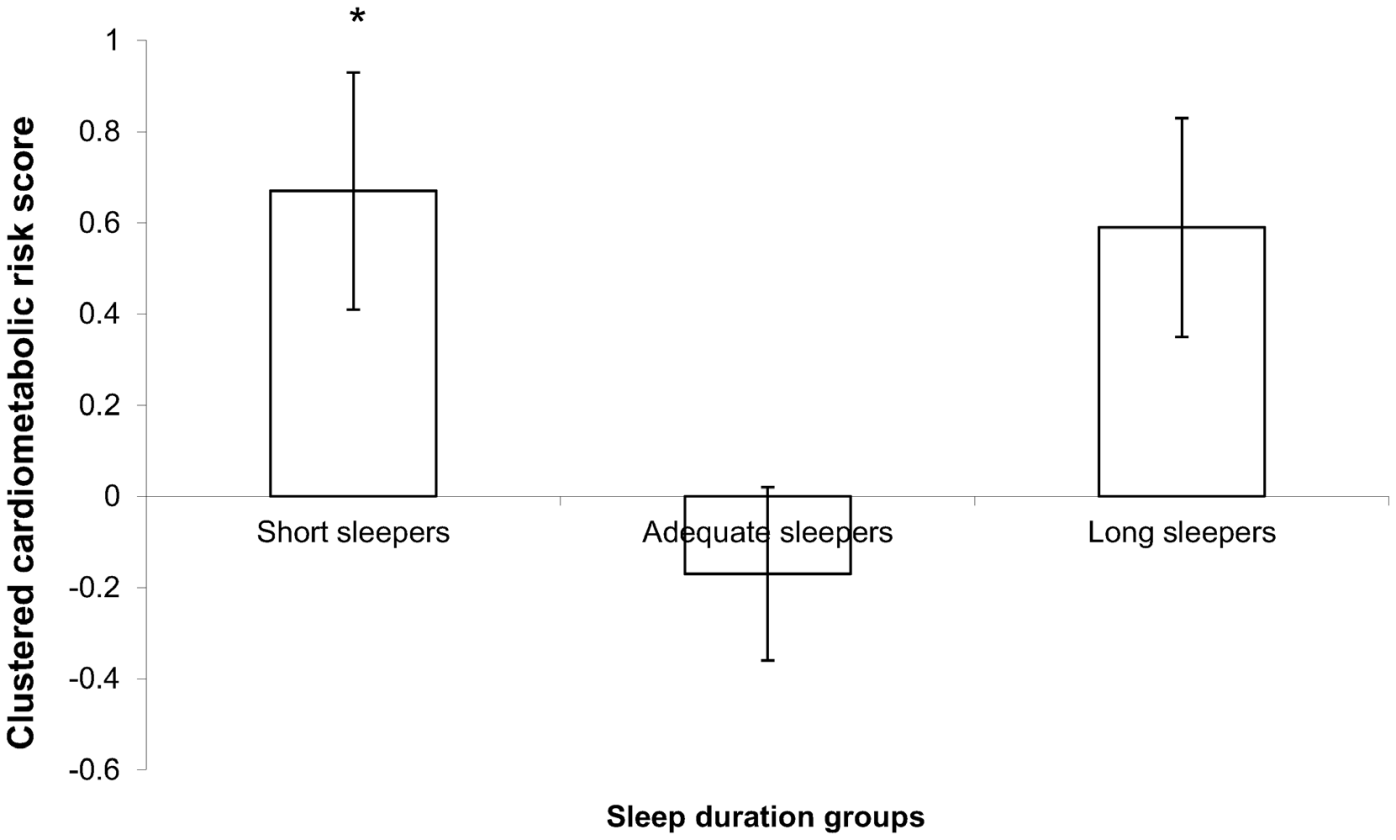
Clustered cardiometabolic risk score between sleep duration groups. *Legend:* Data are presented as mean values with standard errors of the mean. Clustered cardiometabolic risk scores between sleep duration categories were compared by analysis of covariance, followed by a Tukey post-hoc test to contrast mean differences. The model was adjusted for age, sex, smoking habits, highest education level, total annual family income, alcohol consumption, coffee intake, menopausal status, daily caloric intake, and moderate-to-vigorous physical activity as covariates. *P*<0.05 for the ANOVA analysis. *P<0.05 vs. adequate sleepers. Short sleepers (≤6 h of sleep per night; *n* = 90), adequate sleepers (7–8 h of sleep per night; *n* = 571), and long sleepers (≥9 h of sleep per night; *n* = 149).

Finally, clustered cardiometabolic risk scores were compared between sleep duration groups (Figure 1). After adjusting for age, sex, smoking habits, highest educational level, total annual family income, alcohol consumption, coffee intake, menopausal status, daily caloric intake and moderate-to-vigorous physical activity participation, the clustered cardiometabolic risk score was significantly higher in short sleepers compared to adequate sleepers (*P*<0.05).

## Discussion

Collectively, a U-shaped relationship (or inversed J-shaped association) was observed between sleep duration and the prevalence of metabolic syndrome. Sleeping less than 6 h or more than 9 h per night was associated with a greater prevalence of meeting criteria for the metabolic syndrome compared to sleeping 7–8 h a day in adults. After adjustment for possible confounders, only short sleep duration was associated with clustered cardiometabolic risk and an increase in the odds of meeting criteria of the metabolic syndrome. Taken together, these results suggest that long sleep duration does not convincingly increase the risk of being diagnosed with the metabolic syndrome while short sleep duration seems to play an important role. The present results emphasize that short sleeping habits should be regarded as another important risk factor of the metabolic syndrome.

The results of the present study are somewhat concordant with the few studies published to date on the association between sleep duration and the metabolic syndrome. Wu et al. [18] reported that short sleep duration was associated with a higher prevalence of the metabolic syndrome in an apparently healthy population from Taiwan. Likewise, short sleep duration was associated with the presence of metabolic syndrome in other studies [21], [22]. Both short and long sleep durations were also related to an increased risk of the metabolic syndrome and its components in other studies [34], [35]. Similarly, Hall et al. [19] showed that sleep was associated with the metabolic syndrome in a multi-ethnic cohort of midlife women involved in the SWAN Sleep Study. In contrast, Arora et al. [20] reported that long sleep duration was associated with a greater risk of metabolic syndrome in older Chinese. As discussed by the authors, the finding that short sleep duration was unrelated to the metabolic syndrome and its components after adjustment for covariates was not in line with the available evidence and could possibly be explained by an attenuation of the relationship with age, the presence of obstructive sleep apnea (not available in their study) or long sleep being a consequence of ill health.

The mechanisms that underlie the association between short sleep duration and the metabolic syndrome are not well understood but could involve adverse physiologic and hormonal changes, including decreased glucose tolerance, increased insulin resistance, increased sympathetic tone, increased blood pressure, and elevated blood cortisol concentrations as shown in experiments with short-term sleep restriction [3], [4], [9], [17]. Low-grade inflammation is also increased with short sleep, with possible implications not only for cardiovascular risk but also for other chronic conditions including cancer [36]. Lack of sleep has also been shown to increase food intake and contribute to weight gain and obesity [37]. Although self-reported food intake was not significantly different between sleep duration groups in the present study, the abdominal adiposity criterion was met by 39% of short sleepers. Thus, the relationship between shortened sleep duration and cardiometabolic risk could be mediated, in part, by individual associations with obesity, glucose metabolism, and their downstream pathophysiologic consequences. However, future experimental studies will be needed to elucidate the pathways between insufficient sleep and cardiometabolic risk, and to rule out any reverse causation.

Extensive epidemiological evidence suggests that a sleep duration of 7–8 h per night in adults is associated with the maintenance of good health [29]. A growing body of evidence shows that insufficient sleep is associated with mental distress, depression, anxiety, impaired academic performance, weight gain, hypertension, diabetes, high cholesterol levels, premature death, and adverse health behaviors such as physical inactivity and poor eating habits [37]. There is thus a need to more clearly understand the determinants and mechanisms involved in regulating sleep duration and sleep patterns, and to identify high-risk individuals who are in greater need of preventive strategies. Results of this study showed that cigarette smoking, low socio-economic status, coffee intake, and moderate-to-vigorous physical activity participation were all associated with short sleep duration. However, short sleep duration was still associated with an increase in the odds of having the metabolic syndrome after adjusting for these covariates, thus suggesting that the relationship was independent of these covariates and potential confounders.

The present results need to be interpreted in light of the following. First, the direction of causality cannot be determined from cross-sectional data. Second, although good agreement has been found in previous studies between self-reported sleep durations and those obtained through actigraphic monitoring [38], [39], the single question approach does not provide information on sleep quality and our sleep instrument has not been validated. Third, the external generalizability of our findings may be restricted to adults of Western European descent. Finally, there are the commonly acknowledged limitations associated with self-reported food intake and physical activity participation (e.g. recall bias), and the possibility of residual confounding by unmeasured variables is always a possibility in observational studies (e.g. depression, obstructive sleep apnea, insomnia, medications). In particular, the 7–8 h sleep group had higher annual family income and education level; however, adjusting for these two variables does not adequately capture aspects of socio-economic status such as the degree of order or regularity in a home, which is conducive to good sleep hygiene, or neighborhood characteristics.

In summary, the present study provides evidence that short sleep duration is associated with an increased clustered cardiometabolic risk and greater odds of having the metabolic syndrome in adults. Future studies should examine other dimensions of sleep (e.g. sleep quality, timing, architecture, consistency and continuity) which may be related to the metabolic syndrome and its components.
