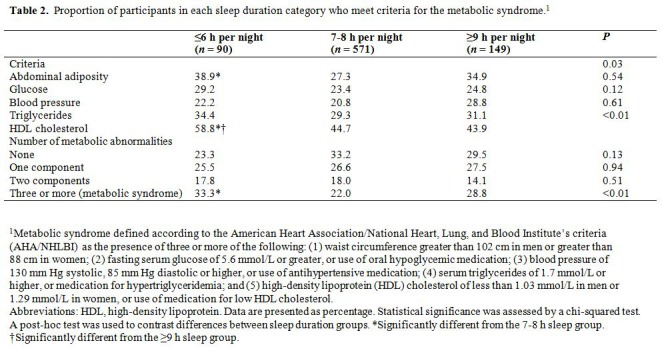# Correction: Seven to Eight Hours of Sleep a Night Is Associated with a Lower Prevalence of the Metabolic Syndrome and Reduced Overall Cardiometabolic Risk in Adults

**DOI:** 10.1371/annotation/1bf80584-08ec-47c2-ba45-4e77554cd50a

**Published:** 2013-10-30

**Authors:** Jean-Philippe Chaput, Jessica McNeil, Jean-Pierre Després, Claude Bouchard, Angelo Tremblay

There was an error in Table 2. The correct version of the table is available here: 

**Figure:**